# Highly selective removal of cationic dye using a novel synthesized polyacrylic polyacrylamide phosphate (PAA@PAm@P) hydrogel

**DOI:** 10.1038/s41598-026-56160-x

**Published:** 2026-06-24

**Authors:** AbdElAziz A. Nayl, Ismail M. Ahmed, Sultan A. Alsahli, Wael A. A. Arafa, Khaled L. AlShammari, Sobhi M. Gomha, Meshari D. Alanazi, Ahmed Salah Doma, Awad F. A. AlHazmi, Stefan Bräse, Ahmed I. Abd-Elhamid

**Affiliations:** 1 Department of Chemistry, College of Science, Jouf University, 72341 Sakaka, Al Jouf Saudi Arabia; 2 Department of Chemistry, Faculty of Science, Islamic University of Madinah, Madinah, Saudi Arabia; 3 Department of Electrical Engineering, College of Engineering, Jouf University, 72388 Sakakah, Saudi Arabia; 4 Polymer Department, Advanced Technology and New Materials Research Institute (ATNMRI), City of Scientific Research and Technological Applications (SRTA-City), New Borg Al-Arab City, Alexandria, 21934 Egypt; 5 Northern Region Cement Co, 8391, 75392 Turaif, Northern Borders Saudi Arabia; 6 Institute of Biological and Chemical Systems – Functional Molecular Systems (IBCS-FMS), Kaiserstrasse 12, 76131 Karlsruhe, Germany; 7 Composites and Nanostructured Materials Research Department, Advanced Technology and New, Materials Research Institute, City of Scientific Research and Technological Applications (SRTA-City), New Borg Al-Arab, Alexandria, 21934 Egypt

**Keywords:** Polyacrylic acid, Polyacrylamide, Phosphate, Hydrogel, Swelling, Methylene blue dye, Environmentally sustainable adsorbent, Chemistry, Environmental sciences, Materials science

## Abstract

In this study, a novel phosphate-functionalized hydrogel adsorbent, poly(acrylic acid-g-polyacrylamide) phosphate (PAA@PAm@P), was successfully synthesized for considerable adsorption of methylene blue (MB)-dye from aquatic solutions. The base hydrogel, poly(acrylic acid-g-polyacrylamide) (PAA@PAm), was prepared via free-radical copolymerization of acrylic acid and acrylamide, followed by mechanical homogenization to obtain hydrogel particles. Surface modification was subsequently achieved through phosphorylation using trisodium phosphate at 180 °C for 4.0 h, yielding the functionalized micron-sized PAA@PAm@P hydrogel. The fabricated materials were characterized using different analytical techniques, such as SEM, FTIR, TGA, and EDS, to confirm successful structural modification and functional groups incorporation. Batch adsorption experiments were conducted to investigate the influence of operational parameters including contact time, adsorbent dose, initial pH, initial MB-dye concentrations, NaCl concentrations, and temperature. The modified micron-sized PAA@PAm@P hydrogel exhibited rapid adsorption kinetics, reaching equilibrium within 15.0 min, which is twice as fast as the unmodified PAA@PAm (30.0 min). The material also demonstrated exceptional swelling behavior with a maximum swelling ratio of 5590% within 3 min, significantly higher than that of PAA@PAm (1712% after 7.0 min). Experimental results indicated that kinetic data were best described by pseudo-second-order (R^2^ = 0.9978) and intra-particle diffusion mechanisms (R^2^ = 0.971) while isotherm data were best fitted well with the Freundlich isotherm model with a remarkably high maximum adsorption capacity of 1000 mg g⁻^1^ for MB-dye. Furthermore, micron-sized PAA@PAm@P hydrogel showed excellent regeneration capability, maintaining high adsorption efficiency over five consecutive adsorption–desorption cycles. Comparative analysis with recently reported hydrogel adsorbents confirmed the superior adsorption performance of the developed material. These findings demonstrate that the phosphate-functionalized PAA@PAm@P hydrogel is a highly efficient, reusable, and environmentally sustainable adsorbent for rapid removal of cationic dyes from contaminated water.

**Supplementary Information:**

The online version contains supplementary material available at 10.1038/s41598-026-56160-x.

## Introduction

Recently, green chemistry has offered promising, sustainable strategies to address major global challenges, particularly freshwater scarcity, which remains a worldwide concern. Accordingly, considerable efforts have been directed to safeguard existing water resources and enable their reuse following appropriate treatment processes^1^. Over the last few years, environmental pollution has increased significantly due to industrial development, technological progress, and rapid population growth, posing a worldwide challenge in providing access to safe and sustainable water resources^2–4^. As a result, water pollution, as an integral aspect of environmental degradation, has become one of the most critical challenges confronting the world today. Annually, significant amounts of colored water containing different types of hazardous chemical materials produced from various industrial activities, including plastics, printing, wool, leather, textiles, and paper, are discharged into the surrounding environment, leading to contaminated water bodies. Consequently, great attention has recently been given to environmental issues, such as eliminating contaminated water containing hazardous chemicals from being released into environmental ecosystems and pure water resources^5,6^. Dyes are among the most hazardous industrial wastes, as many are carcinogenic and toxic. Even at very low concentrations, they can have serious effects on ecological systems and pose significant risks to human health. Nevertheless, the widespread and growing use of dyes across various industrial sectors has significantly contributed to contamination of surface and groundwater resources. Where most such dyes introduce considerable amounts of pollutants into water resources, frequently due to insufficient treatment or uncontrolled release. These dyes, such as Methylene blue (MB)-molecule, which is a cationic dye, are commonly chemically stable and persistent, enabling them to accumulate in aquatic systems and subsequently enter the food chain, where their highly toxicity, carcinogenic, and mutagenic effects pose serious risks to ecosystems and public health^7–10^. Consequently, wastewater produced from these industrial activities should undergo appropriate treatment before being released into the environment. MB-dye is a dye widely used in daily life, which can cause adverse health risks to humans, including vomiting, respiratory difficulties, diarrhea, and nausea. The limitation of MB-molecules by cost-effective adsorbents has been recognized internationally as an effective and environmentally sustainable method, effectively reducing the dye’s impact on the environment^9,11^. Hydrogel-based systems, a rapidly emerging field, have emerged as a promising approach to remove these species from aquatic media owing to their mechanical properties, stimulus–response, high removal efficiency, reusability, operational simplicity, eco-friendly characteristics, and cost-effective performance^12–19^. Also, various approaches, including advanced oxidation processes, chemical degradation, adsorption, and coagulation–flocculation, were investigated and developed to treat water containing dyestuffs. Among these approaches, the adsorption methods are extensively utilized owing to their significant benefits, including ease of design, efficiency, cost-effectiveness, and simplicity^2,20^. Owing to the favorable properties of hydrogels, particularly the large surface area and abundant accessible active sites, which promote effective trapping and adsorbing contaminants, hydrogels exhibit enhanced adsorption performance. Consequently, increasing research efforts have been directed toward the design, development, and optimization of hydrogel-based adsorbent materials to achieve superior sorption capacities for dye species from aquatic media^21^. Despite their unique properties that make hydrogels attractive candidates for pollutant adsorption, they continue to face significant challenges, particularly inadequate mechanical strength. Consequently, these promising works have devoted considerable effort to addressing these limitations by incorporating polyacrylamide into various materials. Recently, numerous studies have examined modified polyacrylamide (PAm)-based hydrogels, demonstrating their superior adsorption efficiency for MB dye from wastewater. Different novel, eco-friendly, effectiveness, and promising PAm-based and other hydrogels adsorbents were investigated, such as polyacrylamide-hexagonal boron nitride nanocomposite hydrogel (PAM/hBN)^22^, sodium alginate-modified polyacrylamide (PAAm/SA)^23^, Polyvinyl alcohol/carboxymethyl cellulose/ZSM-5 zeolite biocomposite (PVA/CMC/ZSM-5 zeolite membranes^24^, carboxymethyl cellulose-graft-poly(acrylamide)/magnetic biochar (CMC-g-poly(AAm)/CL-Fe_3_O_4_)^25^, polyacrylamide/sodium carboxymethyl cellulose/magnetic halloysite nanotube (PAm/CMC/MHNT)^26^, κ-carrageenan/potato starch bio-hydrogel^27^, polyacrylamide-graft-diethylenetriaminepentaacetic acid and chitosan with graphene oxide (APAm/DTPA-CS/GO)^28^, hemicellulose-graft-ployacrylamide (hemi-g-PAm)^29^, carboxymethyl starch co-(polyacrylamide/polyacrylic acid) (CMS co (PAm/ PAA))^30^, pullulan polysaccharide/polyacrylamide/activated carbon (PUL/PAm/GO)^11^, pullulan polysaccharides graft-polyacrylamide-GO (PUL/PAm/GO)^31^, V_2_O_5_-*Gum Ghatti*-Cl-poly (AAM-co-MAA)^32^, poly(acrylic acid)/poly(acrylamide)/calcium hydroxide nanoparticles (PAm/PAA/CHNs)^21^, and other works, to remove MB molecules and other contaminants from aqueous solutions. The surfaces of many reported adsorbent hydrogel materials contain a variety of active functional groups, including –NH₂, –OH, –COOH, –C=O, and –C–O, which provides adsorption capabilities of dye molecules through various mechanisms such as hydrogen bonding, intermolecular interactions, and electrostatic attraction^22–32^. Nevertheless, most of these hydrogel materials still suffer from certain disadvantages including relatively low adsorption capacity, limited mechanical stability, and poor reusability. Such limitations are mainly attributed to inadequate physicochemical characteristics, such as unfavorable morphology, restricted surface area, insufficient thermal stability, and the limited accessibility of functional groups. To address these limitations of PAm-based hydrogels as adsorbents, numerous chemical and physical modification strategies have been investigated by incorporating reinforcing materials into the PAm matrix to fabricate PAm-based hydrogels with improved adsorption performance and enhanced physicochemical stability. To the best of our knowledge, no research work has yet reported the grafting of polyacrylic acid with polyacrylamide and phosphate to produce a novel adsorbent hydrogel aimed at enhancing hydrogel characteristics and adsorption efficiency. Therefore, the main aim of this work lies in the fabrication of a novel micron-sized phosphate-functionalized hydrogel adsobent (PAA@PAm@P). Polyacrylamide phosphate was incorporated to introduce highly reactive phosphate groups that have stronger ion-exchange and electrostatic interactions capabilities toward cationic dyes. Also, the simultaneous presence of carboxyl (PAA), amide (PAm), and phosphate (PAm@P) groups creates synergistic multi-functional adsorption networks with considerable variety of active adsorption sites. Therefore, this investigated tri-functional (PAA@PAm@P) hydrogel structural may enhances adsorption affinity, structural stability, and diffusion accessibility thereby distinguishing the present system from conventional PAA-based adsorbents Accordingly, our work was directed toward fabricating a novel adsorbent, polyacrylic acid-g-polyacrylamide phosphate (PAA@PAm@P) hydrogel via free-radical polymerization, to improve its swelling and adsorption properties and eliminate MB-dye from aquatic media. SEM, FTIR, TGA, and EDS were applied to characterize the modification of the investigated hydrogels. Different parameters affecting the sorption behavior of MB molecules onto the PAA@PAm@P hydrogel were studied. Also, experimental data were analyzed using isotherm, kinetic, and thermodynamic models and compared with recent published works to evaluate the adsorption potential of the prepared hydrogels as a promising material.

## Experimental

### Materials

Acrylic acid (AA) (98%, Acros), Acrylamide (Am) (Spectrum), N, N'-Methylenebisacrylamide (BIS) (99%, Sigma-Aldrich), Potassium persulfate (99%, Sigma-Aldrich), sodium tungstate (99%, Universal Laboratories), Sodium hydroxide (99%, Sigma-Aldrich), trisodium phosphate (99%, Sigma-Aldrich), methylene blue (99%, Sigma-Aldrich), HCl (30%, El Salam for Chemical Industries).

### Preparation of PAA@PAm

To 150 mL bi-distilled water, 2.5 g acrylamide was added under stirring to complete dissolution. Next, 5.0 mL of acrylic acid was added to the previous solution with continuous stirring. Thereafter, 0.25 g Bis and 0.5 g PPs were added to the mixture and stirred to give a clear solution. The temperature of the system was raised to 80 °C till gelatination. The bulk gel was homogenized to yield small gels.

### Preparation of PAA@PAm@P hydrogel

To prepare PAA@PAm@P hydrogel, 8.0 g of Na_3_PO_4_ (P) were dissolved in 1000 mL bi-distilled water, and the temperature was raised to 180 °C for 2.0 h. The obtained gel was filtered, washed with water to remove unreacted materials, froze at − 20 oC and then lyophilized. The produced solid was crushed and sieved (500 µm) to yield a micron-sized PAA@PAm@P hydrogel.

### Characterization

The fabricated hydrogel materials, PAA@PAm and PAA@PAm@P hydrogels, were characterized using the tools discussed in the Supplementary Materials section (“Materials” section).

### Adsorption experiments

The prepared copolymer PAA@PAm@P hydrogel was employed to study the effect of adsorption factors of MB from aqueous solution, such as contact time, adsorbent dose, pH, temperature, initial MB concentration, and NaCl dose. The adsorption investigations were carried out in 100 mL glass beakers containing 50.0 mL of MB-dye aqueous solution. Initial concentrations of MB solutions with different concentrations were prepared by dilution from (1000 mg L^−1^) stock MB standards. The initial pH of the MB solution was controlled by NaOH (0.10 M) and HCl (0.10 M). A specific dose of the adsorbent was added, and the mixture in the beaker was stirred for a specified contact time on a hot plate stirrer. The gel was separated from the solution by filtration, and the final concentrations of MB-dye were measured at a specific λ_max_ (λ_max_ = 665 nm ) using a double-beam UV-spectrophotometer under optimum conditions (Dose = 0.025 g, volume = 50 mL, pH = 7, [MB] = 200 mg/L, t = 15 min, and T = 25 °C), unless otherwise cited.

The adsorption percentage of investigated dye (%R) and adsorption capacity (q_e_, mg/g) were calculated by Eqs. (1) and (2), respectively.

1 $$\mathrm{\%}\mathrm{R}=\frac{\left({\mathrm{C}}_{\mathrm{o}}-{\mathrm{C}}_{\mathrm{t}}\right)}{{\mathrm{C}}_{\mathrm{o}}}\mathrm{x} 100$$

where C_o_ and C_t_ are the initial concentrations and the concentrations of dye at time t, respectively

2 $${\mathrm{q}}_{\mathrm{e}}=\frac{\left({\mathrm{C}}_{\mathrm{o}}-{\mathrm{C}}_{\mathrm{e}}\right)\mathrm{V}}{1000\mathrm{w}}$$

where V is the volume of dye solution (mL), and w is the mass of adsorbent (g).

### Mathematical modeling

Different adsorption models (Kinetics, Isotherm, and thermodynamic) applied in this work were represented in the Supplementary Materials file (“Preparation of PAA@PAm” –“Characterization” sections, respectively) and Table S1.

### Regeneration and reusability of the fabricated PAA@PAm@P hydrogel microstructure

**Scheme 1:**
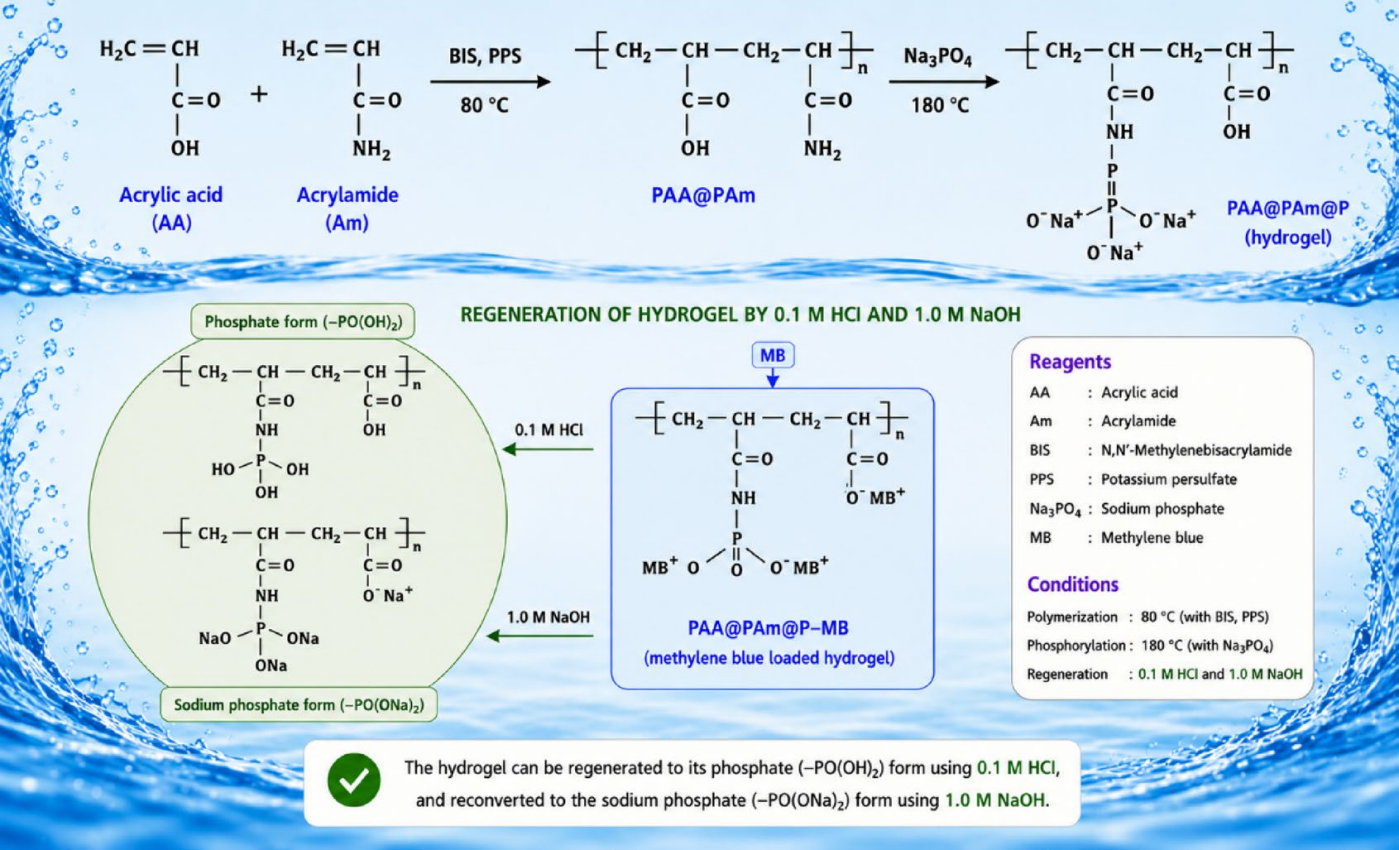
Scheme of the fabrication of micron-sized PAA@PAm@P hydrogel, formation of PAA@PAm@P-MB, and regeneration of the fabricated hydrogel.

To study the regeneration and reusability of the fabricated hydrogel, 0.025 g of micron-sized PAA@PAm@P hydrogel was added to 50 mL of MB dye solution (50 mg/L) of pH 7 and let to stir for 15.0 min at 25 °C. Then, the loaded micron-sized PAA@PAm@P hydrogel was separated by filtration and washed with 10.0 mL 0.1 M HCl for 10.0 min at 25 °C to desorb MB-species. Thereafter, the separated hydrogel washed with 5.0 mL distilled water and further reactivated with 10 mL (1.0 M) NaOH, then washed with 5.0 mL distilled water and adjusted to the next adsorption step. The previous regeneration step was repeated over five regeneration cycles. Scheme 1 summarized the fabrication of micron-sized PAA@PAm@P hydrogel, formation of PAA@PAm@P-MB, and regeneration of the fabricated hydrogel.

## Results and discussion

### Characterization of the fabricated materials

#### SEM

**Fig. 1:**
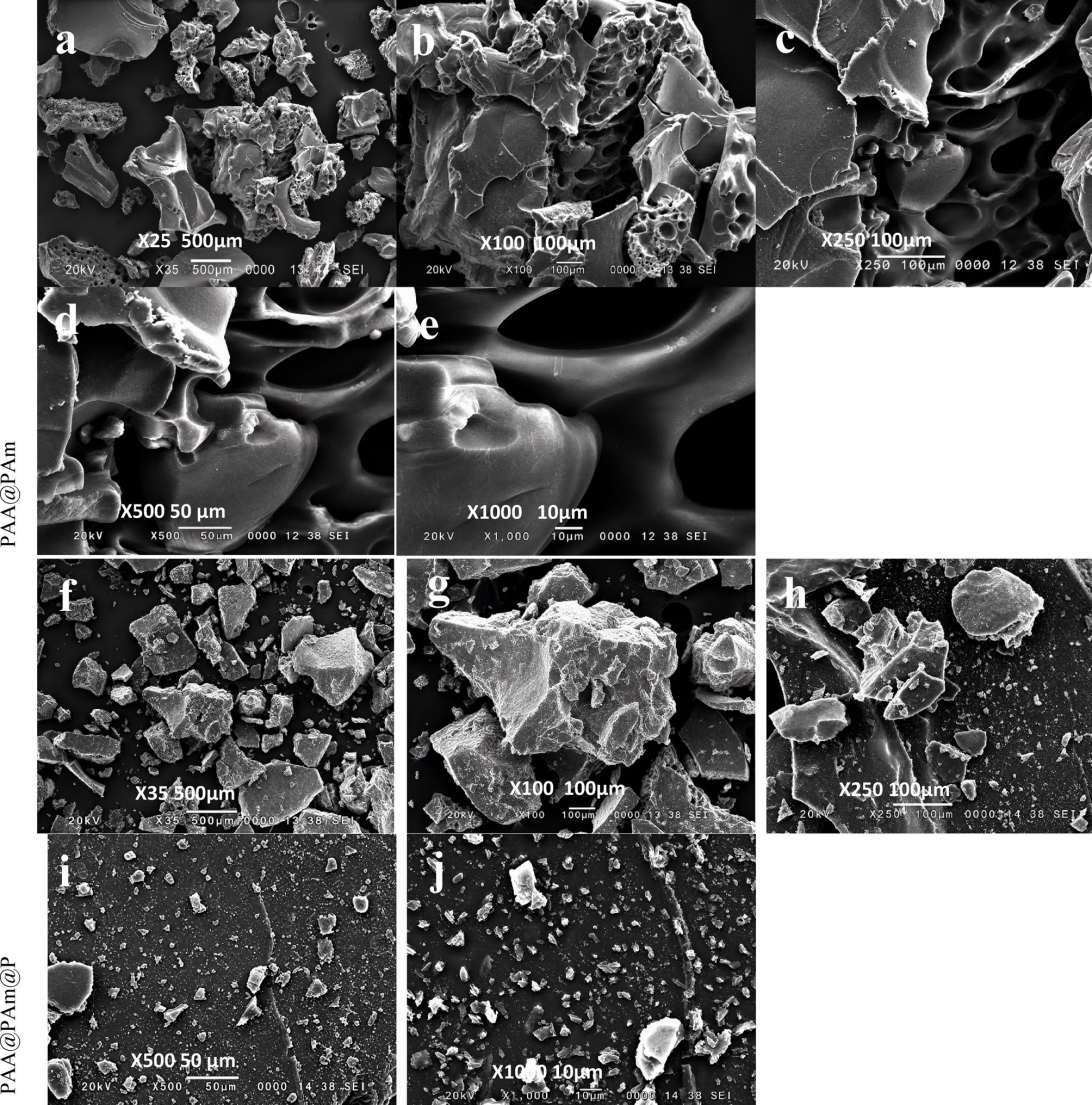

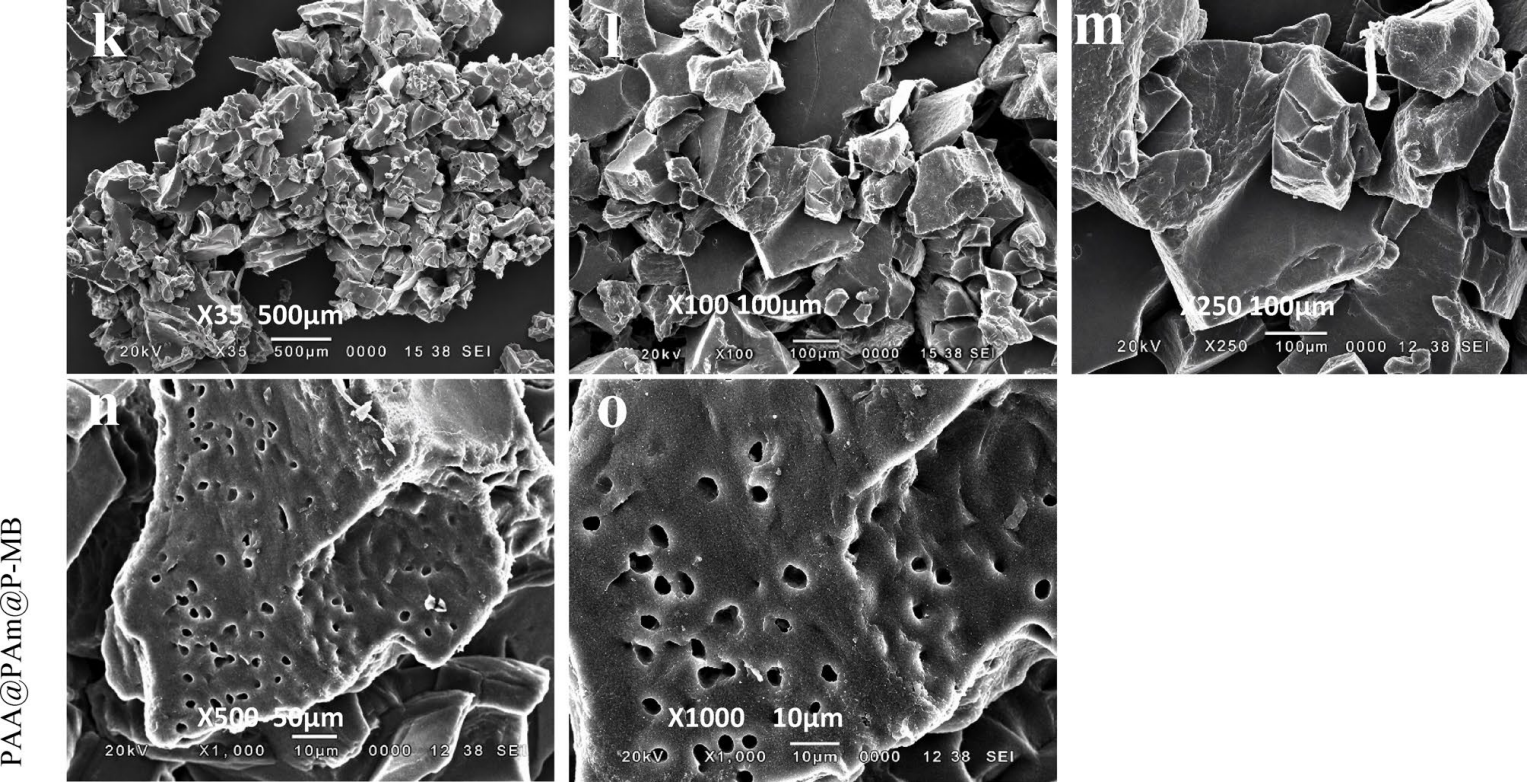
Images of SEM of the PAA@PAm (**a**–**e**), PAA@PAm@P (**f**–**j**), and PAA@PAm@P-MB (**k**–**o**) at different magnifications, respectively.

The SEM is a valuable tool for exploring the surface morphology of fabricated materials. The SEM image of the PAA@PAm is shown in Fig. 1a–o. At low magnification, the PAA@PAm appear as random size and shape, revealing the homogenization effect that was applied to break down the bulk hydrogel into small ones. Thereafter, the broken gel was dried by freeze-drying, and the obtained gels appeared in the SEM image as separated particles. By increasing the magnification power, the pores found in the small gel can be clearly observed, as illustrated in Fig. 1a–e. In addition, it exhibits a homogeneous honeycomb-like porous structure composed of large pores, with the predominant pore size aligning with the condensation-mediated crosslinking between the PAA and PAm backbones. In other words, SEM morphology demonstrated that the prepared PAA@PAm hydrogel possesses a highly porous structure with interconnected pore networks. The cross-sectional SEM images indicates that the internal structure of the PAA@PAm hydrogel is largely dense and compact. Notably, the hemispherical features observed on the cross-sectional surface are most likely associated with the presence of free-water domains within the hydrogel matrix, which generate such textures during the drying process^22^. By treating the PAA@PAm hydrogel with phosphate to form a micron-sized PAA@PAm@P hydrogel and then freeze-dried and meshed (500 µm), the obtained micron-sized gel had random particle size and structure, as represented in Fig. 1f–j. At higher magnification, the prepared micron-sized PAA@PAm@P hydrogel exhibits a uniformly distributed surface morphology throughout the microscale gel, characterized by a well-defined porous structure. These pores are assumed to play a critical role in enabling water and dye molecules diffusion and to provide active sites for interactions with the hydrophilic functional groups of the fabricated PAA@PAm@P hydrogel and causing an improved adsorption capability^6,21,33^. Also, Fig. 1f–j can confirm that phosphate particles were dispersed in PAA@PAm graft copolymer to form PAA@PAm@P hydrogel microstructure. Accordingly, the highly porous micron-sized PAA@PAm@P hydrogel structure can be proven based on the simultaneous electrostatic interactions between phosphate species and the PAA@PAm polymer backbones during the organic crosslinking of PAA chains with PAm (condensation)^22^. After adsorption of MB molecules onto the micron-sized PAA@PAm@P hydrogel and air-drying, the resulting materials rarely aggregate, as shown in Fig. 1k–o. In addition to other pores were observed on the surface of the PAA@PAm@P hydrogel, confirming its stability and the possibility of reusing the fabricated hydrogel. Consequently, the simultaneous electrostatic interaction between phosphate species and the PAA@PAm backbone during the crosslinking process promotes the formation of a dense and highly porous network structures. The resulting wrinkled surface morphology significantly enlarges the accessible surface area for MB-molecules, thereby facilitating stronger adsorbate–adsorbent interactions and improving the adsorption efficiency of the micron-sized PAA@PAm@P hydrogel toward MB dye^22^.

#### FTIR

**Fig. 2:**
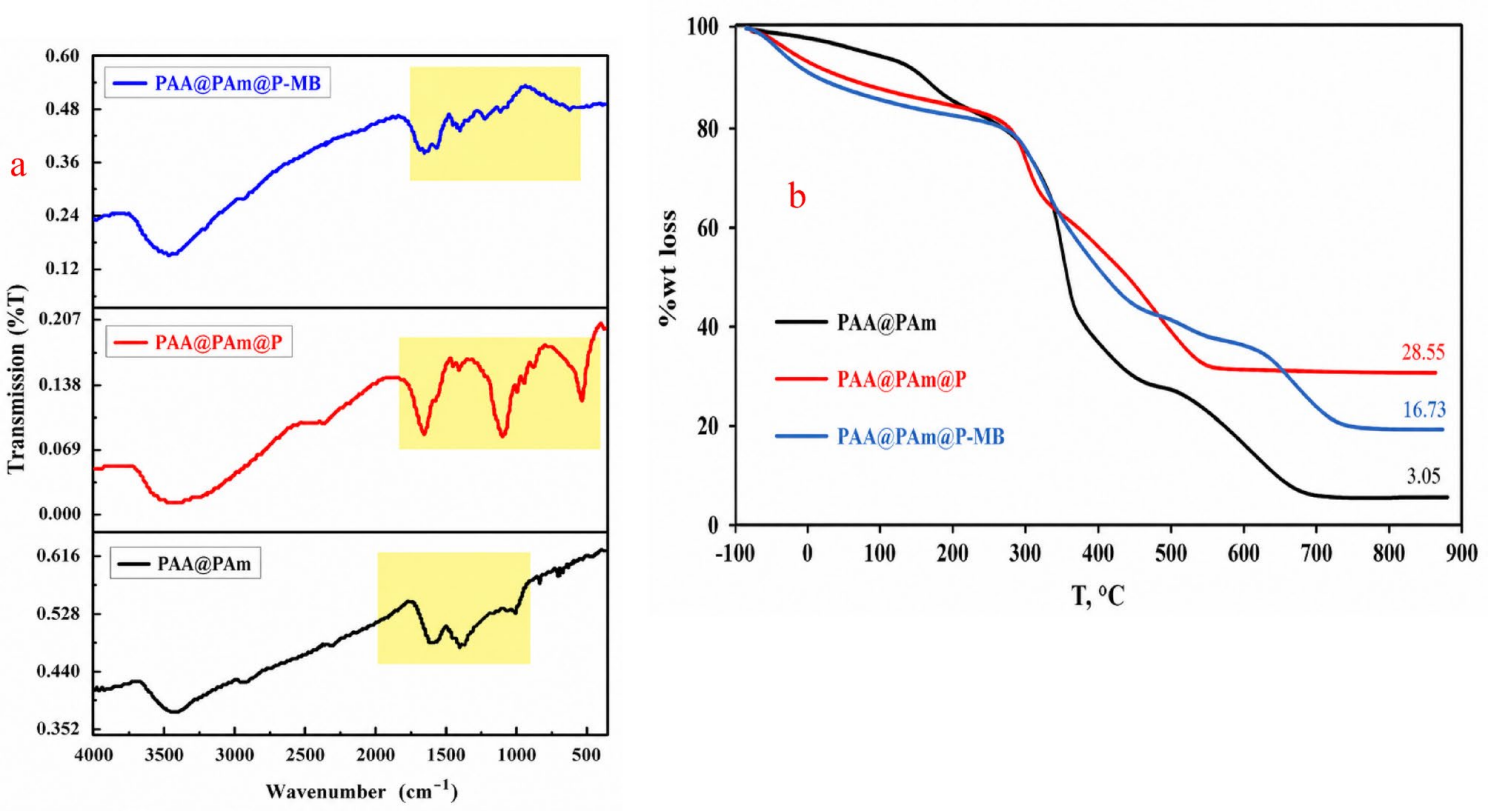
(**a**) FTIR of PAA@PAm, PAA@PAm @P, and PAA@PAm @P-MB, and (**b**) TGA of PAA@PAm, PAA@PAm @P, and PAA@PAm @P-MB.

The FTIR spectra were utilized to give information about the functional groups of the synthesized PAA@PAm, PAA@PAm@P, and PAA@PAm@P-MB composite hydrogels, as presented in Fig. 2a. For the PAA@PAm hydrogel, strong absorbance peaks located at 3419 cm^−1^ can be corresponding to the stretching vibration of OH. In comparison, the peaks at 2953 cm^−1^ correspond to the CH stretching vibration. The characteristic absorbance bands at 1396 cm^-1^ and 1046 cm^-1^ are attributed to the stretching vibrations of C=O and C–O, respectively^34–36^.

These results confirm that the PAA@PAm hydrogel, containing monomer units of PAA and PAm, was successfully fabricated^34^. After modification of PAA@PAm with phosphate, it can be observed that characteristic absorbance peaks shift, with the peak attributed to O–H stretching (3418 cm^−1^) becoming more intense and broader. Moreover, new characteristic absorbance peaks appeared at 1651 cm^−1^, 987 cm^−1^, and 526 cm^−1^, assigned to the stretching vibrations of H_2_O bending, P–O, and O–P–O, which confirm the modification step and the formation of the micron-sized PAA@PAm@P hydrogel^37^. These shifts may be due to the formation of intermolecular H- peaks between the PAA@PAm chains and phosphate species^35^. By mixing PAA@PAm@P with MB dye solution (to form the PAA@PAm@P-MB), absorption peaks at 1650, 987, and 526 cm^−1^ disappeared in the Spectrum of PAA@PAm@P-MB. These variations confirm that MB-species adsorbed onto the fabricated hydrogel.

#### Thermogravimetric analysis (TGA)

The thermal properties of the fabricated PAA@PAm and PAA@PAm@P hydrogels were investigated by TGA to determine the temperatures of glass transition and degradation in addition to other critical polymers properties^36,38,39^. As illustrated in Fig. 2b, the mass loss rates of the two fabricated composites (PAA@PAm and PAA@PAm@P) are notably different, indicating that the addition of phosphate species markedly enhances the thermal stability of the prepared hydrogel. PAA-PAm decomposes over four stages; first stage (28–89 °C; 6.7%) due to evaporation of absorbed surface water. Second stage (89–166 °C; 4.3%) corresponding to liberation of intermolecular water. The lose weight observed in the third stage (166–456 °C; 12.5%) revealed to decomposition of function groups (COOH and CONH_2_). The mass losses observed during the second and third stages of degradation can be owing to the thermal decomposition of the PAA and PAm components^36,40^. The lose weight obtained during the fourth stage (456–800 °C; 45%) can be due to carbonization of the polymeric backbone chain, the residue weight 3.05%^37^. For the fabricated PAA@PAm@P hydrogel microstructure, both absorbed surface water (11%) and intermolecular water (7%) were released at 30–140 °C and 140–288 °C, respectively. Moreover, the decomposition of the function groups was observed at 288–385 °C with weight loss of 21% which may be due to the action of phosphate. The main pyrolysis stage for the fabricated PAA@PAm@P hydrogel was obtained at 385–567 °C with weight loss percent of 32%, and the residue weight percent of 28.5%. This behavior indicates that both PAA@PAm and PAA@PAm@P hydrogels exhibit a considerable degree of thermal stability up to approximately 400 °C^37^. Therefore, TGA is a preferred parameter to evaluate the thermal stability of the fabricated polymers^36,41^. On the other hand, adsorption of MB-dye reduced the amount of the surface absorbed water (8%) and delay the liberation of intermolecular water to 330 °C (13.6) which can be due to the crosslinking effect of the MB among different functional groups. The main degradation step early reached and run out (330–480 °C; 36.7%) compared with micron-sized PAA@PAm@P hydrogel, as shown in Fig. 2b, owing to the degradation of the MB-species adsorbed within PAA@PAm@P hydrogel, Followed by two degradation steps (480–643 °C; 9.4%) and (643–741 °C; 15%) due to pyrolysis function groups and carbon backbone chains.

#### EDS

**Fig. 3:**
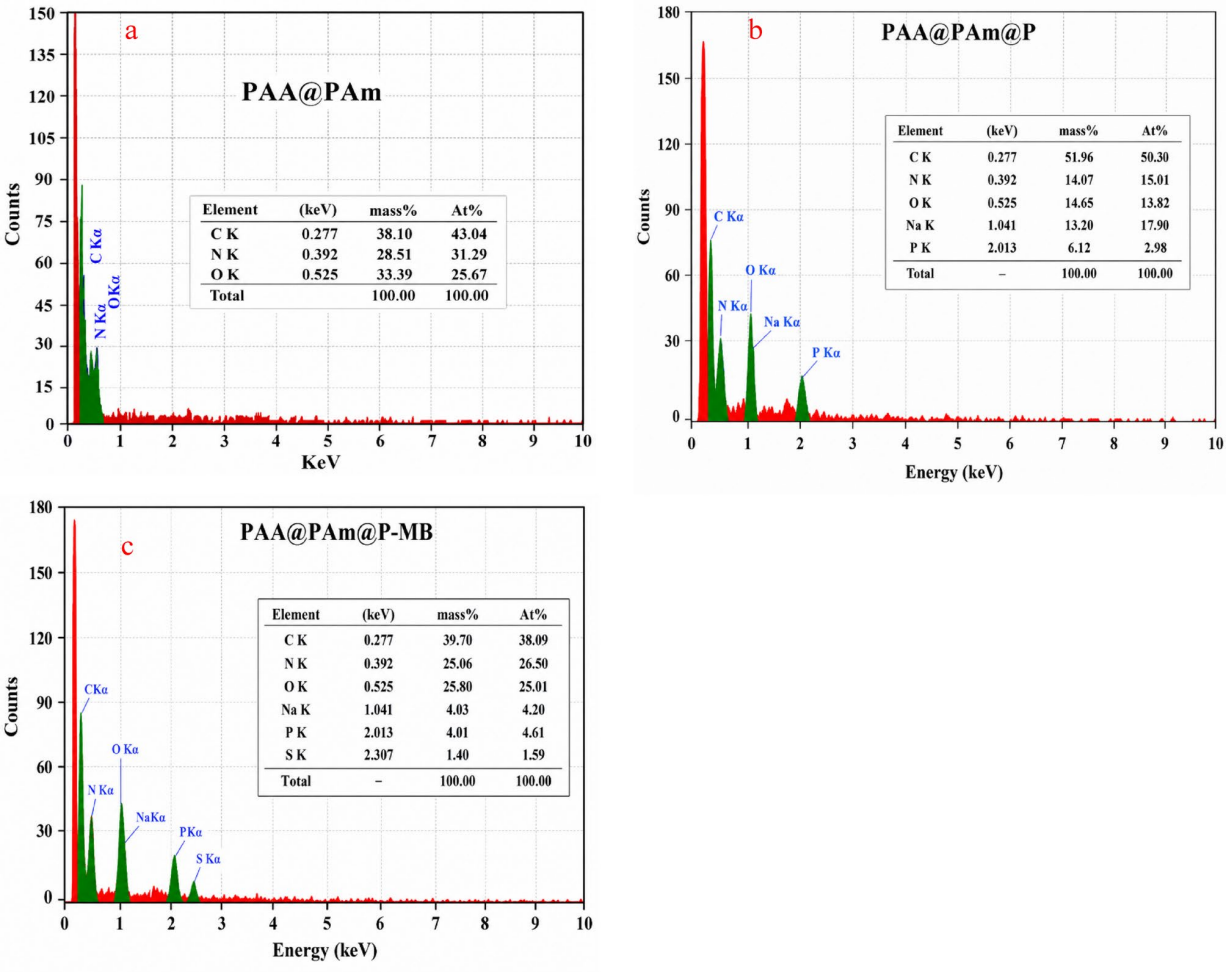
The EDS analyses of (**a**) PAA@PAm, (**b**) PAA@PAm @P, and (**c**) PAA@PAm @P-MB.

EDS is an efficient tool for elemental analysis of the assessed materials. The EDS analysis of PAA@PAm, PAA@PAm@P, and PAA@PAm@P-MB was represented in Fig. 3a–c, respectively. Figure 3a shows the elemental distributions on the surface of the PAA@PAm hydrogel, which is mainly composed of O, C, and N, confirming the high purity of the synthesized material.

After treating with Na_3_PO_4_ to fabricate micron-sized PAA@PAm@P, P and Na appear in the EDS analysis alongside C, O, and N, confirming the successful modification step, as illustrated in Fig. 3b. After the adsorption processes, MB replaces the Na at the active sites, leading to a reduction in the percent of Na in micron-sized PAA@PAm@P hydrogel (At% = 17.9) to (At% = 4.20) in PAA@PAm@P-MB, as shown in Fig. 3c. Moreover, the appearance of sulfur species is associated with the adsorption of MB molecules in PAA@PAm@P.

### Adsorption experiment results

#### Effect of time

**Fig. 4:**
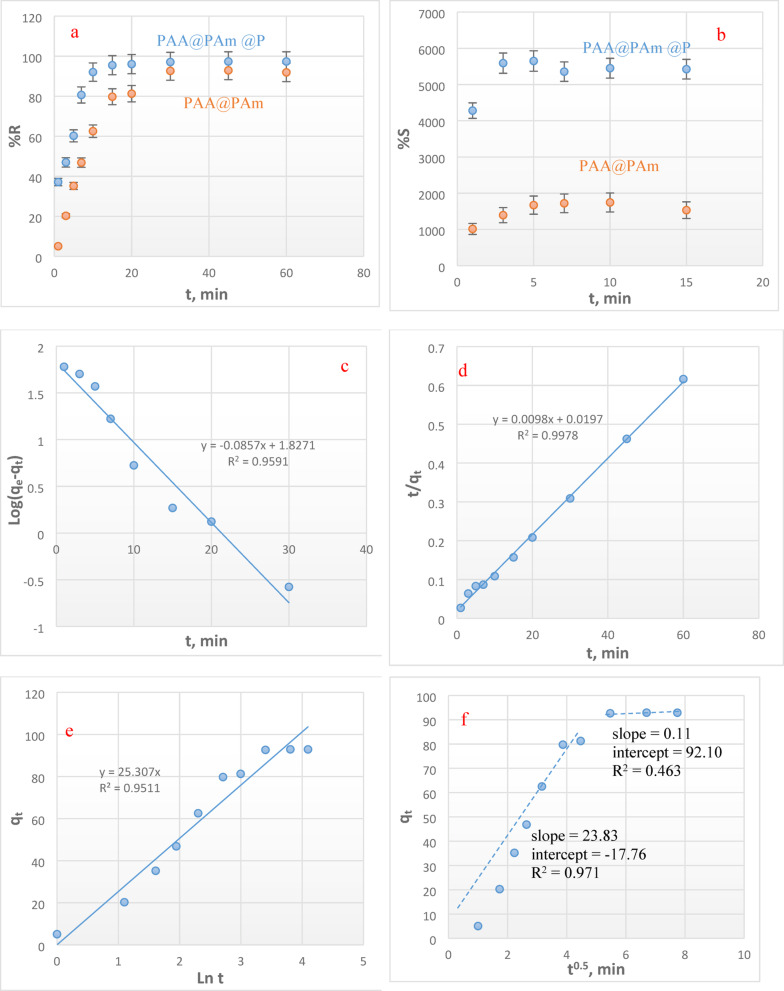

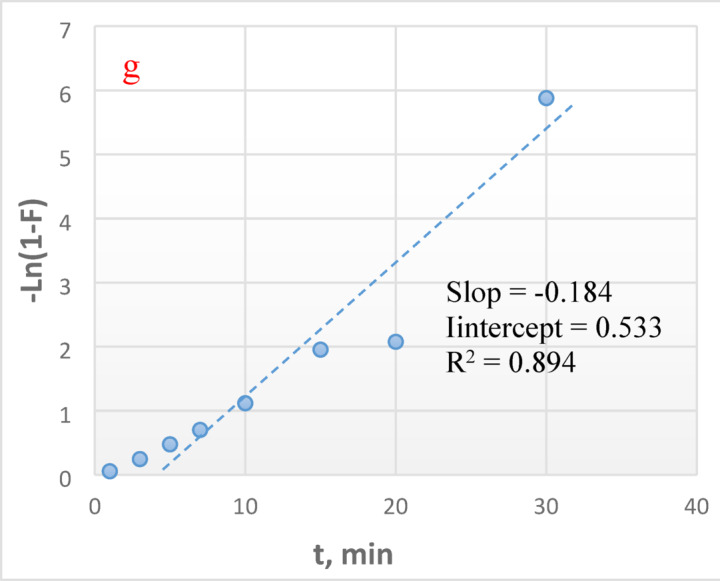
(**a**) Influence of contact time on adsorption percentage of MB-dye onto PAA@PAm and PAA@PAm@P hydrogel, (**b**) swelling capacity of PAA@PAm and PAA@PAm@P hydrogel, The kinetic models, (**c**) pseudo-first-order, (**d**) pseudo-second-order, (**e**) intra-particle diffusion, **(f**) Liquid-film diffusion, and (**g**) Elovich models of MB-dye onto PAA@PAm@P. ([MB] = 50 mg/L, V = 50 mL, Dose = 0.025 g, T = 25 °C, pH = 7).

Contact time is one of the most important factors affecting the cost of the adsorption processes. The influence of contact time on the removal efficiency of MB-species within PAA@PAm and PAA@PAm@P was shown in Fig. 4a. The two materials showed the same behavior: a rapid increase in adsorption rate in the first steps, followed by reaching equilibrium. But to clarify the importance of the modification step, we will explain in detail the effect of the time chart. For PAA@PAm, after 1.0 min, the PAA@PAm showed an adsorption percentage of 5% and increased to reach equilibrium after nearly 30.0 min with adsorption percentage of 92.6%. On the other hand, the micron-sized PAA@PAm@P hydrogel exhibited excellent removal capacity after the first minute, reaching about 37% (about 5 times that of PAA@PAm) and then rapidly increasing to achieve equilibrium after 15.0 min, with an adsorption percentage of 95.5%. The results obtained showed the importance of phosphate modification in reducing the time to reach equilibrium. These adsorption behaviors can be because the active sites on PAA@PAm and PAA@PAm@P hydrogel at the initial time can effectively enhance the attractions of MB-species to adsorbed within the hydrogel lead to fast increase in the adsorption percentages. The adsorption rates become slower between 15.0 min 30.0 min as more active sites are occupied by MB-species. With further time increasing (> 30.0 min), the adsorption equilibrium was reached due to the saturation of active sites within PAA@PAm and PAA@PAm@P by MB-species^21^.

To evaluate the vital role of the phosphate group, the PAA@PAm hydrogel was modified to fabricate the promising micron-sized PAA@PAm@P hydrogel. The swelling percentage as a function of contact time was also investigated, as illustrated in Fig. 4b. The PAA@PAm showed a swelling percentage of %S = 1015 after the first minute, which then slowly increased to %S = 1712 and reached equilibrium after 7.0 min. On the other hand, PAA@PAm@P hydrogel illustrated an excellent swelling ratio of about 4280 after the first minute (about 3 folds of PAA@PAm). It reached a maximum with a swelling ratio of about 5590 (fourfold of PAA@PAm) within 3 min (less than the equilibrium time required for PAA@PAm). This excellent swelling behavior of PAA@PAm@P will promote the rapid arrival of the MB species to the binding sites by diffusion through the hydrogel. Thereafter, it will enhance the adsorption potential and reduce the contact time.

To understand the rate-controlling step and adsorption mechanism of MB species with PAA@PAm@P hydrogel, adsorption kinetics were evaluated utilizing linear and nonlinear regression models^21,34,36,42,43^, such as pseudo-first-order, pseudo-second-order, intra-particle diffusion, liquid-film diffusion, and Elovich as shown in Fig. 4(c, d, e, f, and g), respectively, (as explained in the Supplementary Material file). The kinetic plots are presented in Fig. 4c–g, and the corresponding calculated parameters are summarized in Table 02@. As shown in Fig. 4c,d, the relatively low correlation coefficient (R^2^ = 0.959) for the linear fit indicates that the adsorption of MB dye by PAA@PAm@P hydrogel does not adequately follow the PFO model.

Conversely, pseudo-second-order model exhibits a significantly higher R^2^ (= 0.9978), close to unity, demonstrating that it well describes the experimental data. These findings suggest that the adsorption of MB species within the prepared PAA@PAm@P hydrogel is predominantly governed by chemisorption, involving electron sharing or transfer interactions between MB molecules and the active functional groups of the PAA@PAm@P hydrogel^44,45^. It was observed that the values of q_e. cal_ and q_e. exp_ represented in Table 1 are not equal. This may be attributed to the diffusion stage of MB species within the particles of PAA@PAm@P hydrogel may be involved in the mechanism of the adsorption processes^44^. Therefore, the migration of MB molecules during the adsorption process was assessed by different diffusion models to investigate the rate control steps, as represented in Fig. 4e–g. The results obtained in Fig. 4e indicate that the sorption mechanism of MB-dye within PAA@PAm@P hydrogel may be described also by the intra-particle diffusion model. The adsorption plots display multiple linear regions rather than a single linear relationship, indicating the adsorption processes proceed through more than one kinetic stage. Although the intra-particle diffusion plots are linear, none of the lines pass through the origin, suggesting that intra-particle diffusion contributes to the overall adsorption process but is not the only rate-limiting step. Additional mechanisms, such as complexation or ion-exchange interactions, may also influence the adsorption kinetics^32^. Furthermore, each curve shown in Fig. 4(e and f) consists of two linear regions, confirming that at least two successive steps govern the adsorption processes. The initial linear region corresponds to boundary-layer diffusion, which is associated with the transport of MB molecules through the PAA@PAm@P hydrogel. The second linear region corresponds to a slower, steady-state adsorption phase attributed to intra-particle diffusion of MB-species within the porous structure of the hydrogel. The presence of nonzero intercepts further indicates the existence of a boundary-layer effect. Overall, the observed multi-linearity and boundary-layer thickness suggest that surface adsorption plays a significant role in the MB-dye uptake process, acting in conjunction with intra-particle diffusion rather than as an independent mechanism^46^.

**Table 1:** Parameters of different Kinetic models of MB-species adsorption onto PAA@PAm@P hydrogel (q_e(Exp)_ = 445.5 mg g^−1^).

Kinetic model	Parameters	Values
Pseudo first order	K_1_ q_e_(cal.) (mg g^−1^) R^2^	− 0.20 2.77 0.959
Pseudo-second order	K_2_ q_e_(cal.) (mg g^−1^) R^2^	0.50 102.04 0.9978
Liquid film diffusion	K_fd_ R^2^	− 0.184 0.894
Intraparticle diffusion *Stage (I)* *Stage (II)*	K_i1_ C_1_ R^2^	23.83 − 17.76 0.971
	K_i2_ C_2_ R^2^	0.11 92.10 0.463
Elovich model	ß R^2^	0.038 0.951

**Scheme 2:**
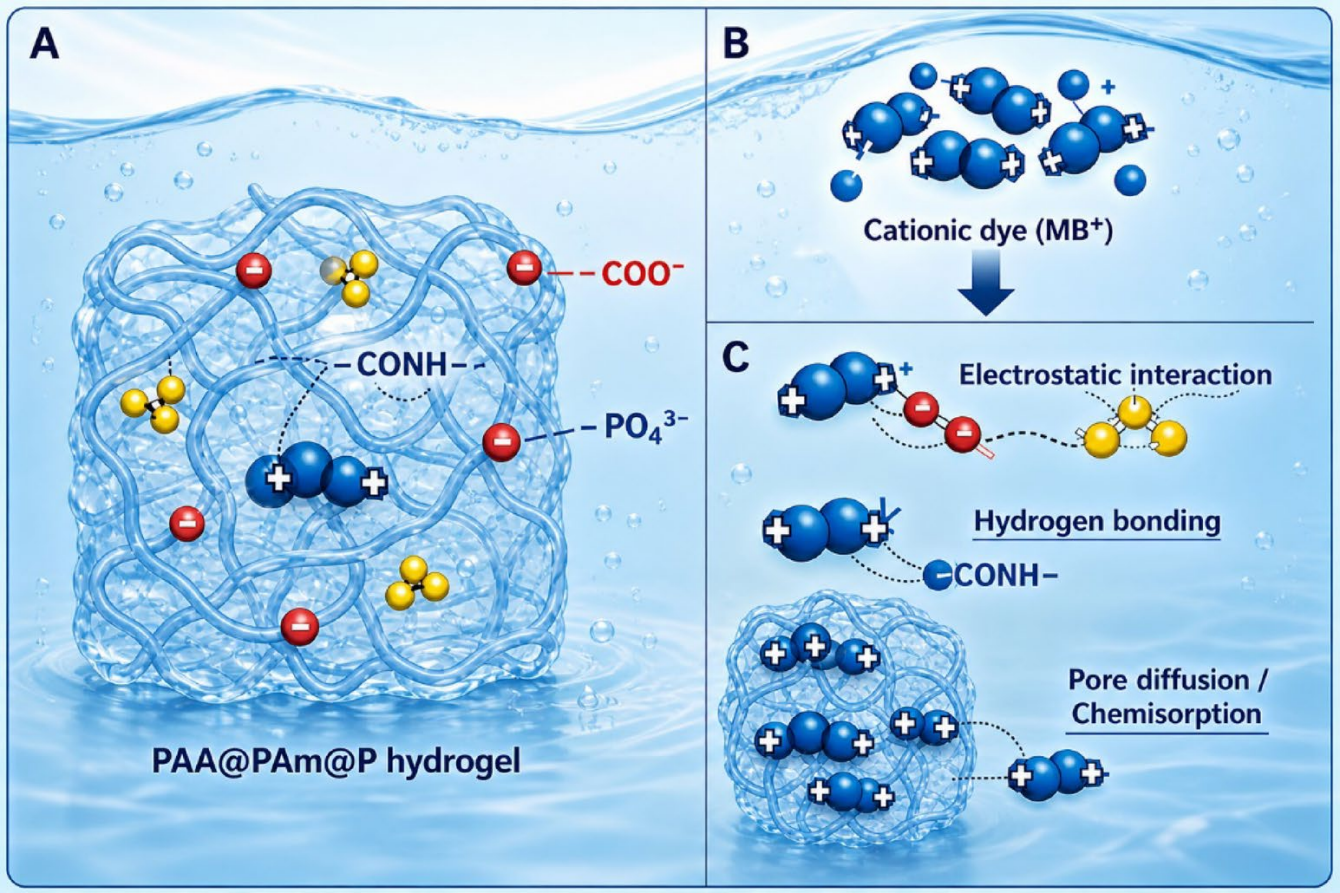
Schematic diagram of the investigated adsorption mechanism; (**A**) PAA@PAm@P hydrogel, (**B**) cationic MB-molecules, and adsorption of MB-dye via (**C**) electrostatic attraction, hydrogen bonding, and pore diffusion with chemisorption.

Based on the obtained results, a proposed mechanism illustrating the adsorption of MB molecules onto micron-sized PAA@PAm@P hydrogel was investigated and represented in Scheme 2. The proposed adsorption mechanism involves multiple interactions including electrostatic attraction between the cationic MB-species and negatively charged functional groups (–COO⁻ and phosphate groups), hydrogen bonding with amide groups (–CONH^–^), and chemical entrapment within the porous of micron-sized PAA@PAm@P hydrogel.

#### Effect of adsorbent dose

**Fig. 5:**
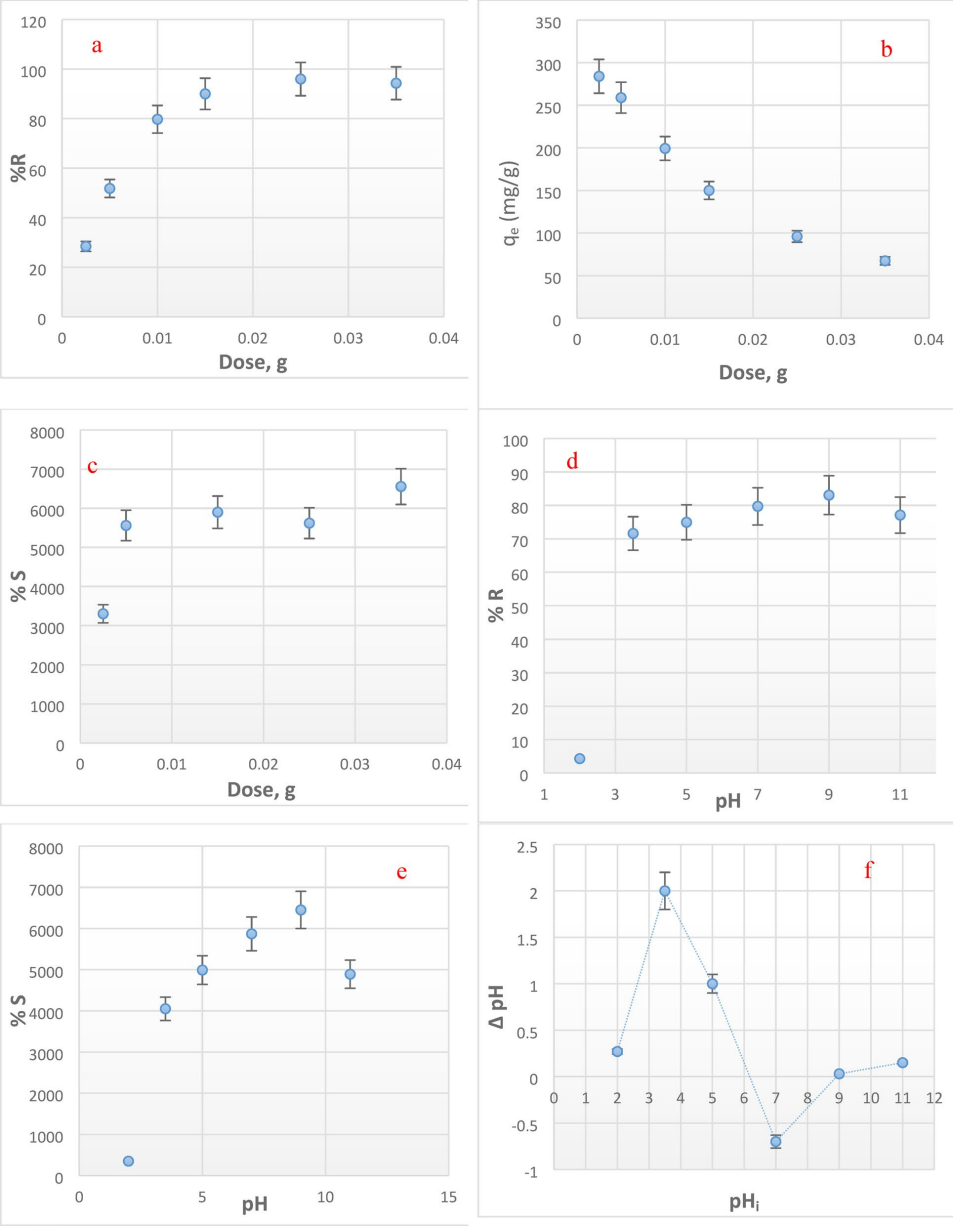
Influence of adsorbent dosage on (**a**) adsorption percentage, (**b**) adsorption capacity (q_e_, mg g⁻^1^) of MB-dye onto PAA@PAm@P, (**c**) swelling percentage ([MB] = 50 mg/L, V = 50 mL, t = 15 min, T = 25 °C, pH = 7). Effect of pH on (**d**) adsorption percentage of MB-dye onto PAA@PAm@P, (**e**) swelling percentage, and (**f**) point of zero charge ([MB] = 50 mg/L, V = 50 mL, t = 15 min, Dose = 0.0.025 g, T = 25 °C).

The effect of PAA@PAm@P hydrogel dose on the adsorption percentage and adsorption capacity (q_e_, mg g⁻^1^) of MB-molecules from aquatic media is plotted in Fig. 5a and b, respectively. As observed, the adsorption percentage (%R) was improved from 28 to 90% with increasing PAA@PAm@P hydrogel dose (0.0025- 0.015/50.0 g mL^−1^). Thereafter, the adsorption percentages slowly increased to 95% and then a plateau was reached with dose of 0.025/50.0 g mL^−1^, then slowly decreased with further increase in the adsorbent dose from 0.025 to 0.035/50.0 g mL^−1^). Also, the adsorption capacity was reduced from 284 to 67.33 mg/g with increasing adsorbent dosage from 0.0025 to 0.035/50.0 g mL^−1^, as shown in Fig. 5b. This behavior is attributed to the fact that, at a constant initial dye concentration, the number of MB molecules in the solution is constant and with increasing adsorbent dosage from 0.0025 to 0.025/50 g mL^−1^, sorption active sites of PAA@PAm@P hydrogel are available to trap MB-species adsorption, facilitating the improvement of adsorption efficiency. With further increasing PAA@PAm@P hydrogel dosage from 0.025 to 0.035/50.0 g mL^−1^, adsorption efficiency would gradually decline owing to more active sites of PAA@PAm@P hydrogel cannot be occupied by MB-molecules, suggesting that the further increasing of micron-sized PAA@PAm@P hydrogel dose suffers from a reduced adsorption efficiency^20,21,47^. On the other hand, increasing the micron-sized PAA@PAm@P hydrogel dose from 0.0025 to 0.005/50.0 g mL^−1^ increased its swelling capacity from 3300 to 5560%. This can be owing to the presence of numerous hydrophilic oxygenated functional groups on the PAA@PAm@P hydrogel, which significantly enhance the compatibility of the prepared hydrogel^43,48^, as demonstrated in Fig. 5c. This increase in the swelling ratio will improve the mobility of the MB dye species through the hydrogel, bringing them closer to the binding site and thereby enhancing the adsorption of MB species within the PAA@PAm@P hydrogel. Next, the swelling ratio remained constant with further increase in the PAA@PAm@P hydrogel dose to 0.035/50.0 g mL^−1^. The nearly constant swelling ratio observed with the further increasing of PAA@PAm@P hydrogel microstructure to 0.035/50.0 g mL^−1^ may be attributed to the limited availability of free H_2_O molecules relative to the increased amount of hydrogel. At this stage, the fixed amount of H_2_O molecules available in the medium becomes insufficient to further hydrate the additional polymeric chains and ionic/phosphate functional groups. Moreover, increased intermolecular interactions and overlapping among micron-sized PAA@PAm@P hydrogel particles at elevated dosage may hinder H_2_O penetration into the internal pores and active hydrophilic sites of the fabricated PAA@PAm@P hydrogel. Consequently, the swelling ratio remained nearly unchanged beyond this dosage^20,42,43^.

#### Effect of initial pH

The initial pH value of the aquatic media is a critical parameter in sorption processes, as it directly affects the surface charges of the sorbent materials and, consequently, the ionization states of functional groups present on the sorbent surfaces. Therefore, the effect of solution pH on the removal behavior of MB-dye from aquatic media within the micron-sized PAA@PAm@P hydrogel was investigated by varying the pH in the range 2–11 and is plotted in Fig. 5d. It can be seen that at pH 2, the adsorption efficiency reached 4%, then rapidly increased to about 71% at pH 3.5, followed by a gradual increase to 83% at pH 9. Finally, with a further increase in the pH value to 11, the %R will decrease to 77%. This is due to, at acidic pH, the binding sites will be occupied by a high number of H^+^ ions. The further increase in the solution’s pH will improve the ionization of the binding groups; consequently, the adsorption percentage will increase significantly. These experimental results can be explained as follows: at pH values below 2.0, the sorption processes exhibited poor efficiency. Conversely, at pH > 3.5, the PAA@PAm@P hydrogel microstructure demonstrated significantly improved sorption performance. This drastic change can be attributed to the surface charge characteristics of the prepared micron-sized PAA@PAm@P hydrogel and the ionization state of the MB-dye. At very low pH (pH 2), the high concentration of H⁺ ions may be led to protonation of active sites groups on PAA@PAm@P hydrogel surface. This positive surface charge induces electrostatic repulsion toward the cationic MB-species, thus limiting their adsorption. As the pH increases to 3.5, gradual deprotonation of PAA@PAm@P hydrogel surface functional groups occurs, generating more negatively charged active sites on the prepared hydrogel surface. This promotes strong electrostatic attraction between these negatively charged adsorption active sites and the cationic MB-species, causing a dramatic improvement in the adsorption capacity^4,43^. In addition, PO_4_^–3^ species have oxygen-containing groups on their surfaces and are negatively charged. Overall, the adsorption of MB molecules is primarily governed by strong electrostatic attraction between the negatively charged functional groups of the micron-sized PAA@PAm@P hydrogel network and the cationic sites of the dye species. The prepared PAA@PAm@P hydrogel contains three types of hydrophilic functional groups, namely –CONH₂, –COO⁻, and –COOH, which exhibit different degrees of hydrophilicity. In this regard, the –COOH and –COO⁻ groups exhibit markedly higher hydrophilicity compared with the amide groups. Besides electrostatic forces, hydrogen bonding may also play a supporting role in the interaction between the PAA@PAm@P hydrogel and cationic MB molecules. These interactions are likely established between the amine functionalities of the MB molecules, and the amide and carboxylic groups present in the micron-sized PAA@PAm@P hydrogel. Despite this contribution, electrostatic attraction remains the principal intermolecular interaction responsible for MB dye uptake in the PAA@PAm@P hydrogel system^31^.

The swelling behavior of the prepared hydrogel was also investigated, as shown in the Fig. 5e. Similar to the adsorption process at low pH, (pH = 2), the hydrogel showed %S = 2350, which rapidly increased to 6450 with a further increase in pH to 9, then decreased to 4890 with a further increase in pH to 11. This is due to, at low pH, only limited swelling ratios are observed, which can be owing to the protonation of the carboxylic groups within the micron-sized PAA@PAm@P hydrogel. This protonation promotes the formation of intra- and intermolecular hydrogen bonds, thereby restricting expansion of the polymeric network. Conversely, with increasing the pH values, the PAA@PAm@P hydrogel demonstrates a substantially higher swelling capacity. This increase is associated with the presence of hydroxyl groups (–OH) and the partial deprotonation of free –COOH functional groups and the hydrophilicity of –COO^−^ and–COOH, leading to enhanced ability of swelling^20,33,46,48^. Therefore the increasing in the swelling capacity values indicate the presence of abundant hydrophilic, oxygen-containing functional groups within PAA@PAm@P hydrogel, which play a crucial role to improve the compatibility of the fabricated hydrogel33, 48].The point of zero charge defined as the pH value at which the binding sites possessed neutral charge (pH = 0). Here, the point of zero charge of the prepared adsorbent (pH_ZPC_ = 6.2), (as shown in Fig. 5f), before this value the binding sites are positively charged and repulsed with cationic dye species. On the other hand, after this value the binding site are negative and attracted with cationic MB species leading to improve the adsorption process. At high basic pH (pH > 10), although the active adsorption sites of micron-sized PAA@PAm@P hydrogel are negatively charged and suitable to adsorb cationic MB-species, the existence of large numbers of sodium cations (Na +) in MB-dye media leading to slightly decreasing in both adsorption and swelling performances of PAA@PAm@P hydrogel system^44,46^. In general, it can conclude that the incorporation of phosphate groups into the polyacrylamide network increases the hydrophilicity of the prepared PAA@PAm@P hydrogel due to the presence of polar and negatively charged phosphate functionalities. This enhanced hydrophilicity improves the swelling behavior and wettability of the fabricated hydrogel, facilitating the diffusion of MB-dye molecules into PAA@PAm@P hydrogel, and increasing the accessibility of active adsorption sites. In addition, the negatively charged phosphate groups promote stronger electrostatic interactions with MB-dye molecules, thereby enhancing the overall adsorption and swelling performances of the investigated PAA@PAm@P hydrogel.

#### Effect of initial MB-dye concentration

**Fig. 6:**
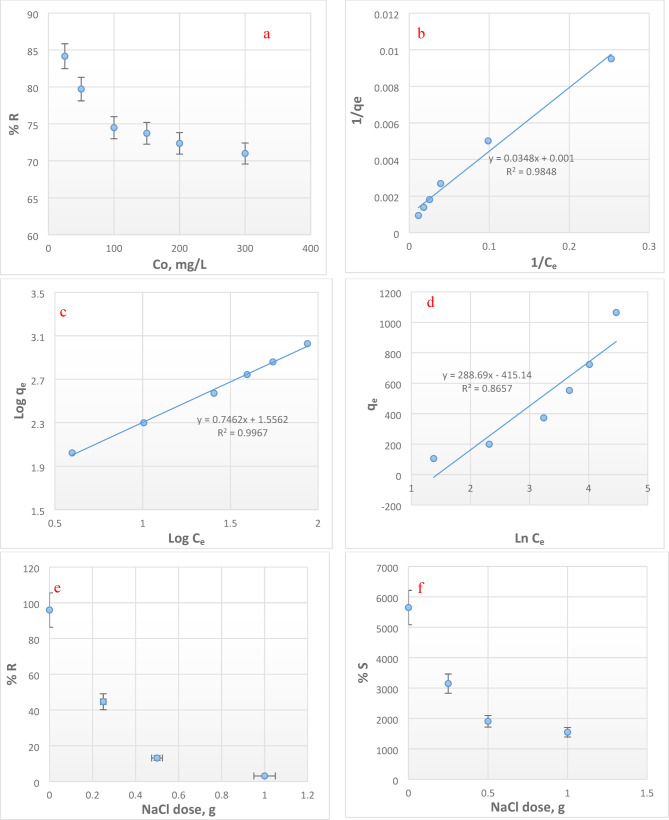
(**a**) Initial concentration, adsorption isotherm, (**b**) Langmuir isotherm plot, (**c**) Freundlich isotherm plot, (**d**) Temkin isotherm (Dose = 0.01 g, V = 50 mL, t = 15 min, T = 25 °C, pH = 7). Effect of NaCl dose on, (**e**) adsorption percentage of MB-molecules by PAA@PAm@P, (**f**) swelling percentage ([MB] = 50 mg/L, Dose = 0.025 g, V = 50 mL, t = 15 min, T = 25 °C, pH = 7).

The impact of initial MB-dye concentration (25–300 mg/L) on the sorption behavior of micron-sized PAA@PAm@P hydrogel was investigated and represented in Fig. 6a. It was observed that with the increase of the initial MB-dye concentration from 25 to 300 mg/L the adsorption performance of the prepared PAA@PAm@P hydrogel was decreased. The maximum adsorption percentage (84%) was achieved at a dye concentration of 25 mg/L, and then decreased to 71% with further increase in MB-dye concentration to 300 mg/L. These findings demonstrate that the initial MB-dye concentrations have negatively impact on the investigated adsorption processes, as the efficiency progressively decreases with increasing concentration, This performance can be due to the saturation and limited availability of active sites on PAA@PAm@P hydrogel at higher MB molecules concentrations or the increased electrostatic repulsive interactions between the adsorbent surface and methylene blue (MB) molecules^43,49^.

The adsorption isotherm was applied to describe the adsorbent-adsorbate relation. Langmuir (Eq. S1), Freundlich (Eq. S2), and Temkin (Eq. S3) isotherms. These models are the more famous isotherm models employed to interpret adsorption experimental results^20,33,48^, as represented in the Supplementary Materials file (“Preparation of PAA@PAm” section). The relation coefficients (R^2^) and the calculated parameters obtained from fitting linear plots in Fig. 6b–d are reported in Table 2. Based on the equilibrium isotherm analysis, the adsorption data for MB-dye species within the micron-sized PAA@PAm@P hydrogel are best described by the Freundlich model, with the highest R^2^ value (≈0.996). This strong fit suggests that MB molecule adsorption proceeds via a multilayer mechanism on a heterogeneous surface. Furthermore, the Freundlich constant *n* exceeds unity, with a corresponding 1/*n* value of 0.77 (< 1), indicating a favorable adsorption process for MB species within the PAA@PAm@P hydrogel^20,25,49^. The calculated Temkin model parameters presented in Table 3 reflects the lower linearity (R^2^ ≈ 0.866) value than that obtained for Langmuir and Freundlich isotherms. Thus, the adsorption of MB species onto PAA@PAm@P hydrogel does not follow Temkin model. On the other hand, for the Temkin model, the adsorption energy parameter b_Ʈ_ (8.58 kJ/mol) has a positive value indicating the adsorption process is exothermic. Furthermore, the low b_T_ value indicates weakness of the interactions between MB-dye and the surface of micron-sized PAA@PAm@P hydrogel^8,20,23,31,34^. The obtained R^2^ values revealed that the Freundlich model has the best correlation to the experimental results than Langmuir and Temkin models.

**Table 2:** Isotherm parameters of the adsorption processes of MB-molecules within PAA@PAm@P hydrogel.

Isotherm model	Parameters	Values
Langmuir	q_o_ (mg/g)	1000
	b (mL/mg)	0.029
	R^2^	0.985
Freundlich	K_f_ (mg/g)	36
	1/n	0.77
	R^2^	0.996
Temkin	A_Ʈ_	4.21
	b_Ʈ_	8.58
	R^2^	0.866

**Table 3:** Thermodynamic parameters of the adsorption of MB-dye by PAA@PAm@P hydrogel.

Dye	T, K	ΔG°, kJ mol^-1^	ΔH°, kJ mol^-1^	ΔS°, J mole^−1^ K ^−1^
MB	303	− 6119.83	− 1.53 × 10^4^	− 30.50
	313	− 5724.36		
	323	− 5405.66		
	333	− 4680.66		

#### Effect of NaCl concentrations

The influences of NaCl dose as an interfering salt on the sorption performance of MB-dye from aquatic media by the PAA@PAm@P hydrogel were illustrated in Fig. 6e. It was observed that the removal capacity of MB-dye decreased from 96 to 3% with increasing NaCl concentration from 0.0 to 0.1 g. This behavior can be attributed to the presence of Na^+^ ions, which screen the binding active site on the micron-sized PAA@PAm@P hydrogel and thereby reduce the adsorption percentage. Besides, the presence of NaCl will reduce the swelling percentages, as shown in Fig. 6f, where the swelling ratio was reduced from 5648 to 1548% with further increase in the NaCl dose in the range of 0.0 -1.0 g. This will reduce the space among the polymer chains, which retard the movement of the aqueous solution through the gel. This behavior can be attributed to the weakening of electrostatic interactions between water molecules and the micron-sized PAA@PAm@P hydrogel due to increased ionic strength. Moreover, the presence of Na⁺ ions induces competitive interactions between salt ions and water molecules for available adsorption sites^48^. As a result, a low number of the MB-dye species will reach the binding site, leading to a reduction in the removal efficiency.

#### Effect of temperature

**Fig.7:**
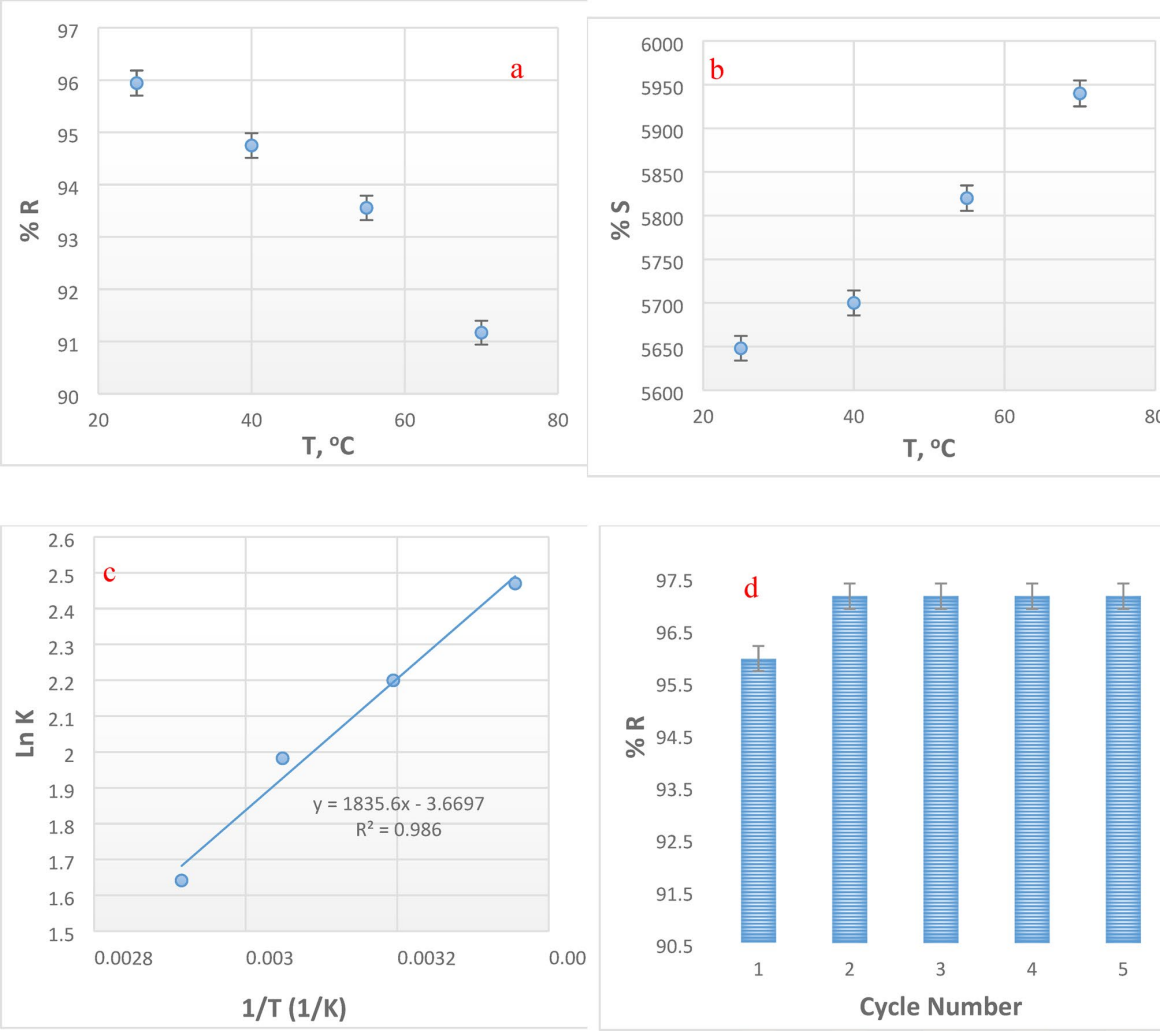
Influence of temperature on (**a**) adsorption percentage of MB-dye by PAA@PAm@P, (**b**) swelling percentage ([MB] = 50 mg/L, Dose = 0.025 g, V = 50 mL, t = 15 min, T = 25 °C, pH = 7). , (**c**) relation between ln K vs 1/T on the adsorption of MB-dye by PAA@PAm@P hydrogel, and d) recyclability of PAA@PAm@P with 0.1 M HCl ([MB] = 50 mg/L, Dose = 0.025 g, V = 50 mL, t = 15 min, T = 25 °C, pH = 7).

The influence of temperature on the adsorption and swelling capacities of the PAA@PAm@P hydrogel was studied over the temperature range of 25–70 °C, as shown in Fig. 7a and b, respectively. The results demonstrated that both the adsorption and swelling performance of the PAA@PAm@P hydrogel decrease with increasing temperature, indicating that MB adsorption is exothermic. The observed reduction in removal efficiency can be attributed to the weakening electrostatic attractions and hydrogen-bonding interactions between the hydrogel adsorbent and MB molecules at higher temperatures^49,50^.

##### Adsorption thermodynamic study

The variation of the thermodynamic factors, such as standard enthalpy (ΔH), standard.

entropy (ΔS), and standard free energy (ΔG), were detected using the Van’t Hoff equation.

By plotting lnKc versus 1/T, the ΔH and ΔS could be calculated from the slopes and intercepts, respectively (as represented in the Supplementary Materials file (“Characterization” section)), as shown in Fig. 7c and listed in Table 3. The negative value of ΔH indicates the exothermic nature of the investigated adsorption processes, which align with strong complexation between the micron-sized PAA@PAm@P hydrogel and MB species. The negative value of ΔS indicates reduced disorder at the solid–liquid interface during MB attraction. The negative ΔG value suggests spontaneous adsorption of MB species within PAA@PAm@P hydrogel and is favorable at low temperature^47,49,50^.

#### Regeneration and reusability of the fabricated micron-sized PAA@PAm@P hydrogel

Regeneration and reusability of adsorbents are essential parameters for assessing the practical feasibility of adsorbents in wastewater treatment applications. An adsorbent with considerable regeneration and reusability contributes substantially to lowering treatment costs and improving process sustainability. Therefore, the long-term effectiveness and economic viability of an adsorbent are fundamentally governed by its efficient regeneration and reuse over multiple cycles^21,34,47,49,50^. Herein, the regeneration and reusability of the fabricated micron-sized PAA@PAm@P hydrogel adsorbent were assessed using diluted HCl (0.1 M) for 10.0 min at 25 °C to desorb the MB-species from the fabricated hydrogel, as represented in Fig. 7d. The fabricated PAA@PAm@P hydrogel demonstrated high reusability. The adsorption/desorption performance remained nearly constant at 97% of its original capacity after 5 cycles. These results indicate that the prepared micron-sized PAA@PAm@P hydrogel adsorbent has excellent recyclability. Furthermore, reactivating the prepared hydrogel with 0.1 M NaOH is a crucial step for activating both phosphate and carboxylic groups. This will be reflected positively in the next adsorption step, enhancing the adsorption percentage compared to the original hydrogel.

### Comparison study

To evaluate the efficiency and adsorption performance of the novel fabricated PAA@PAm@P hydrogel, a recent comparative evaluation of different hydrogel materials was carried out to assess the adsorption performance of the prepared micron-sized PAA@PAm@P hydrogel to remove MB species from aquatic media, as reported in Table 4^11,21–23,25,26,28,30,34,43,48,51–56^. The results demonstrated that the optimized hydrogel exhibits superior performance, indicating its strong potential as a promising and novel hydrogel material for the efficient removal of dye molecules from aqueous solutions.

**Table 4:** Recent comparison of adsorption performance of PAA@PAm@P hydrogel with other hydrogels materials used to remove MB species ^1,11,21–23,25,26,28,30,34,43,48,51–56^.

Adsorbent	q_o_ (mg/g)	MB-dye Conc	Time, min	pH	Temp., ^o^C	No. Reusability	Ref
PUL/PAm/AC	591.4	0.3 g/L	120	8	25		11
PAM/PAA/CHN hydrogel	1,056	0.1 g/L	3d (4320)	7	25	5 HCl & DH_2_O 96%	21
PAM/hBN Nanocomposite Hydrogel	13.5	1.0 g/L	10		25	5 D.L water and ethanol	22
PAM/hBN Nanocomposite Hydrogel	13.5	1.0 g/L	10		25	5 D.L water and ethanol	22
PAm/SA PAm	90.90 23.20	0.09 g/L	2520	7	25		23
CMC-g-poly(AAm) CMC-g-P(AAm)/CL CMC-g-P(AAm)/CL-Fe_3_O_4_	83.11% 92.89% 95.01%	1.0 g/L	60	9	25		25
PAm/CMC/MHNT hydrogel	50.5	0.01 g/L	210	11	25	5 0.1 M HCl 81.58%	26
APAM/DTPA-CS/GO	652.99	0.12 g/L	100	7	30	6 91%	28
CMS co (PAm/PAA	1700	0.25 g/L	120	7	25	4 0.1 mol/L HCl 77%	30
P(NIPAm-coAAc)/MoS_2_	1258	0.64 g/L	360	7	40		34
poly(MAA-co-AAm)/Cl30B poly(MAA-co- AAm)	6.558 1.113	0.01 g/L	60	8	25		43
XG/AA/AAm/GO *XG/AA/AAm*	1000 730.7	0.2 g/L	60	7	25	5 %1.%2 M HCl 86%	48
GG/PAm/RH/Ulva	51.54	0.05 g/L	120	9	25	4	51
PAm	1024	0.01 g/L	120	8.8	25	3	52
PVA-g-PAM	703	4.0 g/L	1200	6	25	5 23%	53
SAG-g-PAm	69.13	7.04 × 10^−7^ g/L	480	10	25		54
SA-PAm SA-PAm-CNC SA-PAm-BC SA-PAm-TOCN	43.1 44.1 47.1 57.1	0.5 g/L	–	7	25	4 distilled water 55%	55
(PAA-coPAm)-DPNR/Ag-TiO_2_	206.42	0.05 g/L	1440	7	25	5 0.5 M NaOH 90%	56
micron-sized PAA@PAm@P hydrogel	1000	0.5 g/L	15	7	25	5 0.1 M HCl 97.5%	This work

## Conclusion

In the present study, a novel and eco-friendly micron-sized adsorbent hydrogel (PAA@PAm@P) was prepared and evaluated for its swelling and adsorption performance in adsorbing MB molecules from aqueous media. SEM, FT-IR, TGA, and EDS techniques were applied to characterize the fabricated hydrogels. The experimental results revealed that incorporating P into PAA@PAm to fabricate micron-sized PAA@PAm@P hydrogel enhanced the swelling and adsorption performances of the prepared adsorbent. Under optimal conditions, the investigated sorption of MB-dye by the prepared hydrogel followed pseudo-second order and the intra-particle diffusion kinetic models and was predominantly governed by chemisorption. On the other hand, the isotherm study showed that the adsorption performance of the fabricated hydrogel was best described by the Freundlich model, suggesting that MB adsorption proceeds via a multilayer mechanism on the heterogeneous surface of the micron-sized PAA@PAm@P hydrogel, with an adsorption capacity of 1000 mg/g. Moreover, the results of regeneration and recyclability indicate that the prepared PAA@PAm@P hydrogel adsorbent exhibits excellent recyclability, with adsorption/desorption performance remaining nearly constant at 97% of its original capacity after 5 cycles. Finally, the comparative study of the investigated hydrogel highlighted its suitability as a promising, sustainable, and eco-friendly hydrogel for the remediation of MB-dye from environmental samples.

## Supplementary Information

Below is the link to the electronic supplementary material.

Supplementary Material 1.

## Data Availability

The authors state that the article contains all pertinent data needed to support the study’s conclusions. The corresponding author can provide more information upon request.
