# Supplementary material for: Highly selective removal of cationic dye using a novel synthesized polyacrylic polyacrylamide phosphate (PAA@PAm@P) hydrogel
Source: Sci Rep. 2026 Jun 24;16:19564. doi: 10.1038/s41598-026-56160-x (PMC13294480; doi:10.1038/s41598-026-56160-x)
Supplement: Supplementary file 1 — Supplementary Material 1. [file 41598_2026_56160_MOESM1_ESM.docx]

**Highly Selective Removal of Cationic Dye using a Novel Synthesized**

**Polyacrylic Polyacrylamide Phosphate (PAA@PAm@P) Hydrogel**

AbdElAziz A. Nayl^a,*^, Ismail M. Ahmed^a^, Sultan A. Alsahli^a^, Wael A. A. Arafa^a^, Khaled L.AlShammari^a^, Sobhi M. Gomha^b^, Meshari D. Alanazi^c^, Ahmed Salah Doma^d^, Awad F. A. AlHazmi^e^, Stefan Bräse^f*^ Ahmed I. Abd-Elhamid^g^

^a^ Department of Chemistry, College of Science, Jouf University, Sakaka 72341, Al Jouf, Saudi Arabia.

^b^ Department of Chemistry, Faculty of Science, Islamic University of Madinah, Madinah, Saudi Arabia. [smgomha@iu.edu.sa](mailto:smgomha@iu.edu.sa)

^c^ Department of Electrical Engineering, College of Engineering, Jouf University, Sakakah, 72388, Saudi Arabia, [mdalsayer@ju.edu.sa](mailto:mdalsayer@ju.edu.sa)

^d^ Polymer Department, Advanced Technology and New Materials Research Institute (ATNMRI), City of Scientific Research and Technological Applications (SRTA-City), New Borg Al-Arab City, Alexandria, 21934, Egypt [ahmed_egypt25@yahoo.com](mailto:ahmed_egypt25@yahoo.com)

^e^ Northern Region Cement Co, Turaif 75392-8391, Northern Borders, Saudi Arabia. [awwadalazeeme@gmail.com](mailto:awwadalazeeme@gmail.com)

^f^ Institute of Biological and Chemical Systems – Functional Molecular Systems (IBCS-FMS), Kaiserstrasse 12, 76131 Karlsruhe, Germany

^g^ Composites and Nanostructured Materials Research Department, Advanced Technology and New, Materials Research Institute, City of Scientific Research and Technological Applications (SRTA-City), New Borg Al-Arab, Alexandria 21934, Egypt; [ahm_ch_ibr@yahoo.com](mailto:ahm_ch_ibr@yahoo.com).

* Correspondence: [aanayel@ju.edu.sa](mailto:aanayel@ju.edu.sa) or [aanayl@yahoo.com](mailto:aanayl@yahoo.com) (A.A.N.); [braese@kit.edu](mailto:braese@kit.edu) (S.B.)

**2. Experimental**

**2.1. Characterization**

The characterization of the as-prepared materials PAm and PAm@W were carried out by Scanning Electron Microscope (SEM, JEOL GSM-6610LV), Fourier Transmission Infra-Red Spectroscopy (FT-IR) (8400s, Shimadzu, Japan) covered the range from 400-4000 cm^-1^. IR spectra of solid samples were detected using KBr disc method. X-Ray Diffraction (XRD) (XRD-7000 Shimadzu, Japan) was utilized to estimate crystalline structure of the prepared nanoparticles and Raman Spectroscopy, EDS was required to study the elemental analysis of the composite before and after the treatment processes. Thermal stabilities of the prepared composite and its complexes with dyes was studied by Thermo-Gravimetric Analyzer (Shimadzu Thermal Gravimetric Analysis (TGA)—50, Japan).

**2.2. Isotherm models**

The adsorption isotherm model was required to ascribe the interaction of the adsorbed species in the liquid phase and the solid phase. Langmuir and Freundlich models were the two famous models which applied. The resulted parameters of the previous models possess valuable information about how the adsorption process occur, nature and affinity of the adsorbent.

**Langmuir isotherm** (1) suggested adsorbent surface with equally energetic adsorbing sites. Its mathematical linear form is written as in Eq. S1:

$\frac{C_{e}}{q_{e}}=\left[\frac{1}{Q^{o}b}\right]+\left[\frac{1}{Q^{o}}\right]C_{e}$ (S1)

Where *q_e_* is the amount of solute sorbed per unit weight of adsorbent (mg/g), *C_e_* is the equilibrium concentration of the solute in the bulk of solution (mg/L), *Q^o^* is the monolayer adsorption capacity (mg/g) and *b* is a constant related to the free energy of adsorption. A plot of *C_e_/q_e_* versus *C_e_* gives a straight line with *Q^o^* and *b* determined from the slope and the intercept, respectively.

**Freundlich isotherm** (1) is an empirical equation based on an exponential distribution of adsorption sites and energies. It is represented as in Eq. S2:

$\ln q_{e}=\ln k_{f}+\frac{1}{n}\ln C_{e}$ (S2)

Where *k_f_* (mg/g) and n are Freundlich constants related to adsorption capacity and adsorption intensity, respectively. A linear plot of ln *q_e_* versus ln *C_e_* confirms the validity of the Freundlich model.

**Temkin model isotherm** contains a factor that explicitly taking into account of adsorbent–adsorbate interactions. The linear equation for the model is given as

q_e_ = B lnA + B ln C_e_  (S3)

where A is the binding constant corresponding to binding energy in L mg^−1^ and B is the heat of adsorption in J mol^−1^.

**2. 3. Adsorption Kinetics**

Pseudo first order kinetic is given by ***Lagergren equation*** [S1] Eq.S4:

$\log(q_{e}- q_{t})=\log q_{e}+\frac{K_{\mathrm{ads}} t}{2.303}$ (S4)

q_t_ is the amount of solute sorbed per mass of sorbent (mg/g) at any time, q_e_ is the amount of sorption at equilibrium time and K_ads_ (min^-1^) is the rate constant of pseudo first order sorption.

$q_{e}=\frac{\left( C_{o}-C_{e} \right)v}{1000w}$ (S5)

v is the volume of dye solution (mL) and w is the dry weight of the adsorbent (g)

$q_{t}=\frac{\left( C_{o}-C_{t} \right)v}{1000w}$ (S6)

where C_t_ is the concentration of the dye (mg/L) at different time intervals.

A pseudo second order kinetic model is explained by ***Ho*** equation [S2], Eq. S7.

$\frac{t}{q_{t}}=\frac{1}{K_{2}q_{e}^{2}}+ \frac{t}{q_{e}}$ (S7)

where *K*_2_ is the pseudo second order rate constant (g mg^-1^min^-1^).

**2.4. Thermodynamic model**

For the calculation of thermodynamic parameters, the following equation was used:

*K_c_= C_ad_/C_e_* (S8)

*∆G^°^ = - RT ln K_c_* (S9)

$Ln K_{c}= \frac{{\Delta S}^{o}}{R}- \frac{{\Delta H}^{o}}{RT}$ (S10)

Where *K_c_* is the equilibrium constant, *C_ad_* is the amount adsorbed on the adsorbent (mg/L) at equilibrium and *C_e_* is the equilibrium concentration in solution (mg/L). *∆G^°^*, *∆H^°^* and *∆S^°^* are changes in Gibbs free energy (kJ/mol), enthalpy change (kJ/mol) and entropy change (J.mol/K), respectively. *R* is the gas concentration (8.314 J/mol/K) and *T* is the absolute temperature (K).

**Table S1.** Linear forms of adsorption models.

| **Study** | **Model** | **Linear form** | **Plot** | **Slope and intercept** | **Ref** |
| --- | --- | --- | --- | --- | --- |
| **kinetic** | Pseudo first order | $\log(q_{e}- q_{t})=\log q_{e}+\frac{K_{\mathrm{ads}}t}{2.303}$ | Log (q_e_- q_t_) vs t | Slope=K_ads_/ 2.303  Intercept =1/ K_2_ q_e_ | Lagergren,1898 |
|  | Pseudo second order | $\frac{t}{q_{t}}=\frac{1}{K_{2}q_{e}^{2}}+\frac{t}{q_{o}}$ | t/q_t_ vs t | Slope= 1/ q_e_  Intercept =1/ K_2_ q_e_ | Ho and McKay, 1998 |
|  | Intra-particle diffusion model | $q_{t}=K_{i}t^{0.5}+C$ | q_t_ vs t^0.5^ | Slope= Ki  Intercept = C | Weber and Morris, 1963 |
|  | Liquid-film diffusion | $-\mathrm{Ln} \left( 1-F \right)=K_{\mathrm{fd}}t$ | Ln (1-F) vs t- | Slope= K_fd_ | Boyd, Adamson, Myers,1947 |
|  | Elovich equation | $q_{t}=\frac{1}{\beta}\ln\left( \alpha\beta\right)+\frac{1}{\beta}\ln t$ | q_t_ vs ln t | Slope= 1/β  Intercept =$\frac{1}{\beta}\ln\left( \alpha\beta\right)$ | Chien and Clayton, 1980 |
| Isotherm | Langmuir | $\frac{C_{e}}{q_{e}}=\left( \frac{1}{q_{o}b} \right)+\left( \frac{1}{q_{o}} \right)C_{e}$ | C_e_/q_e_ vs C_e_ | Slope= 1/q_o_  Intercept =1/q_o_ b | Langmuir, 1918 |
|  | Freundlich | $\log q_{e}=\log K_{f}+\frac{1}{n}\log C_{e}$ | log q_e_ vs log C_e_ | Slope=1**/**n  Intercept = logK_f_ | Freundlich, 1906 |
|  | Temkin | $q_{e}=BlnA_{T}+B{lnC}_{e}$ | q_e_ Vs ${lnC}_{e}$ | Slope=B  Intercept = $BlnA_{T}$ | Temkin, 1941 |
|  | Flory–Huggins adsorption | $\log\left( \frac{\theta}{C_{o}} \right)=\log\left( K_{a} \right)+nlog(1-\theta)$ | $\log\left( \frac{\theta}{C_{o}} \right) vs log(-\theta)$ | Slope=n  Intercept = logK_a_ | Horsfall and Spiff, 2005 |
| Thermodynamic |  | $\mathrm{Ln}K_{d}= \frac{\Delta S^{o}}{R}- \frac{\Delta H^{o}}{\mathrm{RT}}$ | Ln Kd vs 1/T | Slope= ΔH°/R  Intercept = ΔS°/R |  |
|  |  | ∆G° = - RT ln Kc |  |  |  |

References

S1. Lagergren, S. About the Theory of So-Called Adsorption of Soluble Substances. Kungliga Svenska Vetenskapsakademiens 526 Handlingar, 24 (1898) 1 – 39.

S2. Ho, Y.S.; McKay, G. The sorption of lead (II) ions on peat. Water Res. 33 (1999) 578 – 584. https://doi.org/10.1016/S0043-528 1354(98)00207-3.

S3. Weber, W.J. and Morris, J.C. Kinetics of Adsorption on Carbon from Solutions. Journal of the Sanitary Engineering Division, 89 (1963) 31-39.

S4. Boyd [G.E.](https://pubmed.ncbi.nlm.nih.gov/?term=BOYD+GE&cauthor_id=20270838) , Adamson [A.W](https://pubmed.ncbi.nlm.nih.gov/?term=ADAMSON+AW&cauthor_id=20270838)., MYERS Jr [L.S.](https://pubmed.ncbi.nlm.nih.gov/?term=MYERS+LS+Jr&cauthor_id=20270838) , The exchange adsorption of ions from aqueous solutions by organic zeolites; kinetics, J Am Chem Soc, 69(11) (1947) 2836-48. doi: 10.1021/ja01203a066.

S5. Chien, S.H. and Clayton, W.R. Application of Elovich Equation to the Kinetics of Phosphate Release and Sorption in Soils. Soil Science Society of America Journal, 44 (1980) 265-268.

<http://dx.doi.org/10.2136/sssaj1980.03615995004400020013x>

S6. Langmuir, I. The Adsorption of Gases on Plane Surface of Glass, Mica and Olatinum. Journal of the American Chemical Society, 40 (1918)1361-1403. <http://dx.doi.org/10.1021/ja02242a004>

S7. Freundlich, H.M. Over the Adsorption in Solution. Journal of Physical Chemistry A, 57 (1906) 385-470.

S8. Temkin, M. I. “Adsorption Equilibrium and the Kinetics of Processes on Nonhomogeneous Surfaces and in the Interaction between Adsorbed Molecules,” Zhurnal Fiziche- skoi Khimii, Vol. 15 (1941) 296-332.

S9. Horsfall, M. and Spiff, I.A. Effects of Temperature on the Sorption of Pb2+ and Cd2+ from Aqueous Solution by Caladium Bicolor (Wild Cocoyam) Biomass. Electronic Journal of Biotechnology,8 (2005) 1010-1013. <https://doi.org/10.2225/vol8-issue2-fulltext-4>
